# The Outcomes of Surgical and Nonsurgical Treatment in Patients With Spinal Metastases of Lung Cancer: Protocol for a Prospective Cohort Study

**DOI:** 10.2196/38273

**Published:** 2023-01-30

**Authors:** Panpan Hu, Shuheng Zhai, Xiaoxie Liu, Hongling Chu, Feng Wei

**Affiliations:** 1 Department of Orthopedics Peking University Third Hospital Beijing China; 2 Engineering Research Center of Bone and Joint Precision Medicine Peking University Third Hospital Beijing China; 3 Beijing Key Laboratory of Spinal Disease Research Peking University Third Hospital Beijing China; 4 Department of Rehabilitation Peking University Third Hospital Beijing China; 5 Research Center of Clinical Epidemiology Peking University Third Hospital Beijing China

**Keywords:** spinal metastasis of lung cancer, spinal metastases of lung cancer, lung cancer, surgery, overall survival, prediction model, protocol

## Abstract

**Background:**

Spinal metastases of lung cancer (SMLC) usually have a high degree of malignancy and require multimodality treatment. Patients with SMLC who experience clinical symptoms (eg, local pain, emerging or potential spinal instability, and progressive neurological dysfunction) require surgical treatment. However, there are discrepancies in the comparison of outcomes between surgical treatment and nonsurgical treatment.

**Objective:**

This paper presents the protocol for a study that aims to compare the clinical outcomes of surgical treatment and nonsurgical treatment for SMLC, explore the prognostic factors of SMLC, and establish a survival prediction model based on these prognostic factors.

**Methods:**

This is a prospective cohort study, with an anticipated sample size of 240 patients (120 patients in the surgical group and 120 patients in the nonsurgical group). We will collect baseline data, including demographic, clinical, and radiological information, as well as data from patient-reported questionnaires. Patients will be followed up at 3, 6, 12, and 24 months after treatment, and survival status will be assessed every 3 months. The primary outcome is the overall survival period. Prognostic factors associated with overall survival will be analyzed by univariate and multivariate Cox proportional hazards regression. Odds ratios with 95% CIs will be presented. Statistical significance is set at *P*<.05.

**Results:**

This study has been approved by our institute’s Medical Science Research Ethics Committee (IRB00006761-M2021085) after a careful audit of the design and content. Patient enrollment began in June 2022 at our hospital. Data collection is expected to be completed by early 2026, and the study results will be published by mid-2027.

**Conclusions:**

In this study, we propose to set up a prospective cohort of patients with SMLC to investigate the outcomes between surgical treatment and nonsurgical treatment. We will explore the role of surgical treatment in SMLC and provide guidance to peer surgeons.

**Trial Registration:**

Chinese Clinical Trial Registry, ChiCTR2100048151; http://www.chictr.org.cn/showproj.aspx?proj=129450

**International Registered Report Identifier (IRRID):**

DERR1-10.2196/38273

## Introduction

### Background

The effective integration of therapeutic modalities into the treatment of metastatic lung cancer on the spine is a source of debate. Lung cancer is a moderate to high malignancy, and metastasis to the spine usually represents the end stage of the disease [1,2]. According to the scoring systems for spinal metastasis by Tomita et al [3] and Tokuhashi et al [4], patients with spinal metastases of lung cancer (SMLC) are expected to live less than 1 year. Thus, more conservative treatments have been given in the past. In recent years, advances in radiation therapy and systemic antitumor drugs, such as stereotactic body radiotherapy (SBRT), target therapies, and immunological therapies have demonstrated encouraging therapeutic outcomes and have largely expanded the survival time of patients with SMLC [5-11]. At the same time, surgical treatment has been widely accepted for some SMLC cases [12]. A comprehensive treatment, composed of antitumor drugs, radiotherapy, and surgery, has been the current state-of-the-art therapeutic modality for SMLC.

Generally, patients with SMLC experiencing the following symptoms are referred for surgery: severe and refractory local pain, emerging or potential spinal instability, and progressive neurological dysfunction [8,13-17]. After the surgery, adjuvant therapies, including radiotherapy and systemic drugs, are undertaken. Most researchers have stated that adjuvant radiotherapy should be completed around 6 weeks after the operation to spare time for physical recovery and avoid problems related to wound healing [18]; yet, this is difficult to do in a real-world setting [19]. Additionally, previous studies have described discrepancies in the efficacy of surgical treatment for patients with SMLC [20]. According to these works, the median survival time after surgery was reported to range from 2 to 14 months [21]. Because of limitations that included a retrospective study design, no control group, etc, the conclusions of related studies lack high-quality evidence.

### Objective

In our study, a prospective, specific disease cohort of SMLC will be used to compare the effectiveness of surgical treatment and nonsurgical treatment. This study will provide useful information to explore the role of surgical treatment in comprehensive SMLC treatment and develop suitable treatment guidelines.

The primary aim of this study is to investigate and compare treatment outcomes in patients with SMLC. In addition, we also plan to analyze various prognostic factors of SMLC, such as treatment modalities, tumor subtypes, pre- and postoperative neurological function, and more.

## Methods

### Study Design

This study is a prospective cohort study. We will compare the treatment outcomes of surgical and nonsurgical methods for SMLC in a real-world clinical scenario. The study will enroll 240 patients (120 patients in the surgical group and 120 patients in the nonsurgical group) from June 1, 2022, to June 30, 2027. The patients will be visited postoperatively at 3, 6, 12, and 24 months. The study is due to be completed on June 30, 2029.

### Sample Size and Study Timeline

In this study, we plan to compare the overall survival (OS) time between the two groups and explore its prognostic factors. The sample size calculation was based on the survival time and estimation of influencing factors. According to an unpublished retrospective study performed at our center and previous literature [20,21], the average OS time was 11 (SD 4) months in the surgical group and 9 (SD 3) months in the nonsurgical group. Assuming an overall Type 1 error (α) of .05 and Type II error (β) of .1, we need 66 patients in each group. Additionally, there are 12 predictive factors we plan to analyze in the prediction model. According to the sample size estimation method for multivariable regression models, 100 cases in each group are needed (the ratio of surgical to nonsurgical group is 1:1). Considering a final follow-up rate of 80%, a minimum of 120 patients will be required for each group. There were approximately 25 patients undergoing surgery per year at our center; therefore, we plan to enroll the patients over the next 5 years. Since more patients are treated nonsurgically than surgically, all nonsurgical patients treated during the first 5 years will be enrolled.

The study duration is estimated to be 7 years to ensure that all recruited patients are followed up for 2 years. Each visit’s timeline and tasks are presented in Multimedia Appendix 1.

### Patient Enrollment and Group Allocation

**Figure 1:**
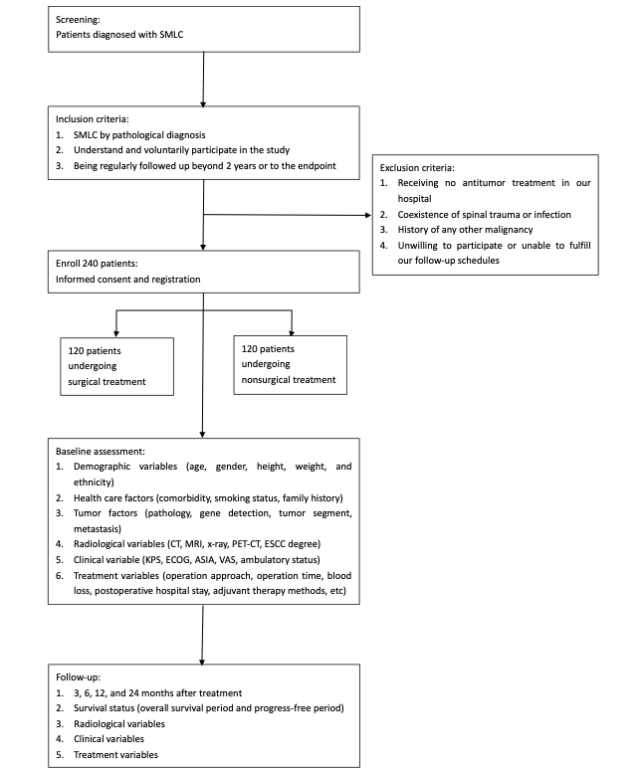
Study flowchart. ASIA: American Spinal Injury Association; CT: computed tomography; ECOG: Eastern Cooperative Oncology Group; ESCC: esophageal squamous cell carcinoma; KPS: Karnofsky Performance Scale; MRI: magnetic resonance imaging; PET-CT: positron emission tomography–computerized tomography; SMLC: spinal metastases of lung cancer; VAS: visual analogue scale.

All patients with SMLC being treated at our center are potential candidates, and those who meet the inclusion and exclusion criteria will be eligible for enrollment in our study. Patients who receive surgical treatment along with postoperative systemic comprehensive treatment will be enrolled in the surgical group. Patients who receive only conservative treatment (eg, radiotherapy, chemotherapy, targeted molecular therapy, immunological therapy, etc) without surgical treatment will be enrolled in the nonsurgical group. All study procedures, as well as the participants’ rights, responsibilities, benefits, and risks, will be communicated, and participants will sign an informed consent form. Figure 1 displays the study flowchart.

In order to participate, patients will need to meet certain eligibility criteria. The inclusion criteria are (1) a pathological diagnosis of SMLC, (2) understanding and voluntarily participating in the study, and (3) being regularly followed up beyond 2 years or to the endpoint. Exclusion criteria include: (1) not receiving antitumor treatment at our hospital, (2) coexistence of spinal trauma or infection, (3) history of other malignancies, and (4) unwillingness to participate or inability to adhere to our follow-up schedule.

### Treatment

A surgical decision is made following the advice of our institutional multidisciplinary treatment team for spinal metastasis. There are 3 indications for surgery at our center: severe yet drug-resistant local pain, progressive neurological dysfunction, and emerging or latent spinal instability. Otherwise, patients are referred for nonsurgical treatment.

In the surgical group, patients are given surgical treatment, neurological rehabilitation, and adjuvant therapies (ie, SBRT and antitumor drugs). In this setting, we have established a synchronized workflow for surgery-based multimodality therapy instead of the conventional step-by-step schedule. A synchronized workflow means that neurological rehabilitation is incorporated and commenced right before the surgery and that SBRT and antitumor drugs are started early and simultaneously during the process of rehabilitation.

In the nonsurgical treatment group, patients are treated with adjuvant therapies exclusively, including radiotherapy, chemotherapy, targeted therapy, immunological therapy, etc. Considering the existence of various adjuvant modalities, this study will only enroll patients who receive both locoregional radiotherapy and antitumor drugs.

### Outcomes

#### Primary Outcome

The primary outcome metric of the study is the OS period. OS is defined as the time after the start of any antitumor therapy for SMLC to death of any cause.

#### Secondary Outcomes

Secondary outcomes include the following:

1. The progress-free survival period, defined as the time after the start of antitumor therapies until disease progression.
2. Recovery of SMLC-related symptoms, defined as the relief of locoregional pain, which is measured by visual analogue scale (VAS) values, and the recovery of neurological status, which is assessed using the American Spinal Injury Association (ASIA) score. Immediate improvement in VAS and ASIA scores is assessed before discharge.
3. Recovery of self-care performance, assessed using the Karnofsky Performance Scale (KPS) score and the Eastern Cooperative Oncology Group (ECOG) performance score.
4. Quality of daily life, assessed by the Short Form 36 Health Survey Questionnaire.

Other outcomes for evaluation include complications in antitumor treatment. Surgical complications are rated according to the system proposed by Clavien et al [22], whereas toxicity of radiotherapy and antitumor drugs is graded using the National Cancer Institute’s Common Toxicity Criteria for Adverse Events (version 5.0). The endpoint of the study is the death of the recruited patient. The primary outcome is assessed by the endpoint of the study, whereas secondary and other outcome metrics are assessed at each visit.

#### Exposure Measures

The exposure measures of interest include demographic variables, clinical data, and treatment-related factors. Demographic variables include age, gender, height, weight, and ethnicity. Clinical data are composed of health care factors (comorbidity, smoking status, family history of tumors); tumor factors (pathology, gene detection, tumor segments, and metastatic status); radiological variables (computerized tomography, magnetic resonance imaging, x-ray, positron emission tomography–computerized tomography, and degree of epidural spinal cord compression); and clinical variables, which include onset time, general status (KPS, ECOG score), neurological function (ASIA score), pain (VAS value), and ambulatory status. Treatment-related factors include operation approach, operation time, blood loss, postoperative hospital stay, adjuvant therapies, etc.

### Data Management and Supervision

We will assign a specific investigation team to assess outcomes and process the data, and train the team in this regard. In addition, we will formulate some basic rules for the process of patient follow-up; outcomes assessment; and data entry, coding, and storage, namely double assessment, dual entry, and checks by independent investigators. Outcome assessors will be blinded to the patients’ intervention allocation. Our institute’s scientific research supervising team, which excludes all investigators in this study, will monitor the study process, data safety and authenticity, and occurrence of adverse events.

### Statistical Analysis

Statistical analysis will be performed using SPSS for Windows (version 20; IBM Corp). The Lilliefors test will be used to examine whether the data are normally distributed. Data will be presented as percentages, mean (SD), or median (range) accordingly. The 1-tailed unpaired Student *t* test and Pearson *χ*^2^ test (or the Fisher exact test) will be used to make comparisons between different groups. The OS and progress-free survival curves will be plotted using the Kaplan-Meier method, and a comparison of the two groups will be performed via the log-rank test. The influencing factors will be analyzed by univariate and multivariate Cox proportional hazards regression. Statistical significance is set at *P*<.05. The survival prediction model will be established by multivariate Cox regression models and the Kaplan-Meier survival curves. The predictive ability of the final model will be evaluated using the Harrell concordance index (C-index) and calibration curves.

### Ethics Approval

This study, which will be conducted according to the Declaration of Helsinki, has been approved by the Medical Science Research Ethics Committee of the Peking University Third Hospital (IRB00006761-M2021085) after a careful audit of the design and content.

### Informed Consent and Funding

We will fully inform the patients of the purpose and procedures of this study, as well as their obligations and potential costs and risks. An informed consent form has been drafted and will be signed voluntarily by each participant.

This study is a self-organized project and receives no financial support or funding from any commercial entity. Therefore, conduct of the study, data processing, and interpretation of the results will not be influenced by any commercial entity.

## Results

Study design, ethical review, and research registration have been completed. Enrollment commenced in June 2022, and enrolled patients are being visited regularly. Patient enrollment is expected to be completed by June 2027, and the study results will be submitted for publication in early 2029.

## Discussion

### Expected Findings

We anticipate that the findings of our cohort study will support the use of a surgical treatment strategy for patients with SMLC. Although we understand that a combination of different antitumor therapies, namely comprehensive treatment, is recommended for patients with SMLC, there is no consensus or established guideline on how to practice it. Our study aims to explore surgery-based comprehensive treatment for patients with SMLC. Through this study, we aim to validate our proposed synchronized schedule for a multimodality therapy and eventually hope to establish a survival prediction model.

### Previous Studies

Fundamentally, the primary goals of surgery are to restore spinal stability, relieve pain, and decompress tumor mass squeezing of the spinal cord, if any. Yao et al [20] reviewed studies on spinal metastasis and found that surgery could significantly improve neurological function and quality of life, even for patients with advanced lung cancer. However, we have not yet gained firm evidence on the impact of surgery on patients’ survival, though many papers have reported favorable postoperation outcomes. Truong et al [17] recruited patients with SMLC undergoing surgery and found that 14 (16%) out of 87 patients survived beyond 12 months. Chen et al [23] reported the median survival of their cohort to be 12 months; patients undergoing excisional surgery had a longer survival time than those with palliative surgery. Contrarily, in a systematic review by Armstrong et al [21], the authors reported unfavorable outcomes for surgical intervention, of which a shorter median OS was found in the surgical group compared to the nonsurgical group (7.5 months vs 8.5 months). This result was echoed in Amelot et al’s [24] study. These authors concluded that survival of SMLC depends merely on genotype and neurological and personal status, and scarcely on surgical resection. In the past decade, the combination of separation surgery and high-dose hypofractionated SBRT has emerged as an effective option for spinal metastasis, including SMLC [18]. The core principle of this procedure is to circumferentially remove the tumor mass encasing the spinal cord and create a safe zone between the cord and the tumor (≥3mm) for the subsequent delivery of radiation treatment [9,25,26].

As our understanding of the molecular biology of lung cancer increases, many mutated targets are being identified, including the epidermal growth factor receptor (EGFR), anaplastic lymphoma kinase, ROS1, BRAF V600E mutation, etc [6,7,27,28]. Targeted drugs can act directly on these specific mutations and diminish tumor cells. Dohzono et al [29] compared the median OS of patients with SMLC receiving or not receiving EGFR tyrosine kinase inhibitors and found the former group enjoyed a longer OS period (21.4 months vs 6.1 months). In recent years, the application of some immunotherapeutic drugs has proven to be effective [7]. The concept of immunotherapy is to introduce specific blockers to inhibit some immunological signaling paths that are related to the development and metastasis of tumors. Currently, anti-PD-1 and anti-PD-L1 antibodies have been recognized as the choice of care for some advanced lung cancers after the failure of first-line cytotoxic therapy [30]. Borghaei et al [28] found that the median OS period of PD-L1(+) patients who received specific targeted blockers was longer than that of PD-L1(–) patients (17.7 months vs 10.5 months). Furthermore, systematic use of bone-targeted agents (eg, bisphosphonates and denosumab) has significantly reduced devastating spinal-related events, such as paralysis and loss of self-care ability [31].

### Strengths and Limitations

The main strength of our study is that it is the first research project to examine the feasibility and outcomes of synchronized adjuvant therapies after surgical intervention for SMLC in a prospective cohort and using a more scientific methodology. This methodological merit will strongly empower the justification for using positive yet invasive treatment modalities instead of conventional palliative care in patients with SMLC. Second, for the purpose of rapid recovery, we are starting rehabilitative intervention at an early stage after surgery. In addition, we recognize the importance of starting adjuvant therapies early after surgery, and this practice will be implemented and examined in our clinical trial.

There are some potential biases in this study that might compromise the reliability and accuracy of the results, but we have designed some solutions to reduce bias. First, though it is difficult to blind the intervention providers and patients, outcome assessors and data processors will be blinded. Second, during the follow-ups, we will encourage face-to-face interviews, but online interviews will also be accepted, considering the possible emergence of scenarios like a COVID-19 surge, in order to reduce the rate of dropout.

### Conclusions

We present the protocol for a prospective cohort study of patients with SMLC. Through this study, we will explore the outcomes of surgery-based comprehensive treatment. In addition, we will also investigate prognostic factors of SMLC and provide references for peer surgeons.

## Appendix

Multimedia Appendix 1 The visit timeline and list of tasks.

## Abbreviations

ASIA: American Spinal Injury Association
C-index: concordance index
ECOG: Eastern Cooperative Oncology Group
EGFR: epidermal growth factor receptor
KPS: Karnofsky Performance Scale
OS: overall survival
SBRT: stereotactic body radiotherapy
SMLC: spinal metastases of lung cancer
VAS: visual analogue scale

## Data Availability

The data sets generated during and/or analyzed during this study will be available from the corresponding author upon reasonable request.
