# Supplementary material for: The Outcomes of Surgical and Nonsurgical Treatment in Patients With Spinal Metastases of Lung Cancer: Protocol for a Prospective Cohort Study
Source: JMIR Res Protoc. 2023 Jan 30;12:e38273. doi: 10.2196/38273 (PMC9926339; doi:10.2196/38273)
Supplement: Multimedia Appendix 1 [file resprot_v12i1e38273_app1.docx]

**Multimedia Appendix 1** The visit timeline and list of tasks

| **Tasks list** | Pre-enrollment  -1 month to day -1 | Allocation Day 0 | Treatment period Day 1 to Month 1 | Follow-up period | |
| --- | --- | --- | --- | --- | --- |
|  |  |  |  | Routine follow-ups  Month 3, 6, 12, 24 | Survival follow-ups  Every 3 months |
| Eligibility screen |  | / | / | / | / |
| Informed consent |  | / | / | / | / |
| Allocation | / |  | / | / | / |
| Demographics |  | / | / | / | / |
| Physical examination |  | / | / |  | / |
| Blood routine |  | / | / |  | / |
| Serum chemistry |  | / | / |  | / |
| Serum tumor markers |  | / | / |  | / |
| Imaging examinations |  | / | / |  | / |
| Intervention | / | / |  | / | / |
| Survival status (OS and PFS) | / | / | / | / |  |
| Self-care performance |  | / | / |  | / |
| Neurological status |  | / | / |  | / |
| Patient-reported outcomes | / | / |  |  | / |
| Complications | / | / |  |  | / |
| A cell with “/” inside means the task is not required at this visit.  OS, overall survival; PFS, progress-free survival. | | | | | |
